# Pulmonary Rehabilitation and Asthma

**DOI:** 10.3389/fphar.2020.00542

**Published:** 2020-05-06

**Authors:** Elisabetta Zampogna, Martina Zappa, Antonio Spanevello, Dina Visca

**Affiliations:** ^1^Istituti Clinici Scientifici Maugeri IRCCS, Respiratory Rehabilitation of the Istitute of Tradate (VA), Tradate, Italy; ^2^Department of Medicine and Surgery, Respiratory Diseases, University of Insubria, Varese-Como, Italy

**Keywords:** pulmonary rehabilitation, asthma, exercise training, chronic disease, treatment

## Abstract

Asthma is a chronic inflammatory disease characterized by airflow limitation and variable respiratory symptoms. It is characterized by variable symptoms such as cough, wheeze, chest tightness, and shortness of breath which vary in intensity and time. In order to reach a comprehensive approach of disease management, the importance of non-pharmacological treatment in addition to pharmacological therapy has been recently highlighted. Studies have documented that pulmonary rehabilitation has beneficial effects in patients with asthma, at any stage of the disease, improving exercise capacity, asthma control, and quality of life and reducing wheezing, anxiety, depression, and bronchial inflammation. Although several evidences suggest a role of pulmonary rehabilitation in patients with asthma, additional information is required to identify a specific program in order to improve clinical care based on specific patient’s needs.

## Introduction

Chronic airway inflammation and bronchial hyperresponsiveness typically identify the pathogenesis of asthma disease. These result in the presence of variable respiratory symptoms due to episodic and reversible airflow limitation (National Asthma Education and Prevention Program, 2007; Global Initiative for Asthma, 2018). Asthmatic patients may complain of poor quality of life (QoL) because their daily life activities may be limited by worsening of respiratory symptoms on exertion. Exertional dyspnea is a variable symptom in asthma, in terms of intensity and duration and could be multifactorial (Carson et al., 2013): ventilator limitation, gas transfer abnormalities, pulmonary vascular and cardiac dysfunction, limb muscle dysfunction, and comorbid impairments may contribute individually or in association. Another significant problem is represented by exercise-induced bronchoconstriction (EIB) consisting of bronchoconstriction onset, occurring during or immediately after exercise (Satta, 2000; Storms, 2005; Lucas and Platts-Mills, 2005). In addition, oral steroid therapy taken on a regular basis or occasionally to treat acute exacerbation can lead to steroid-induced myopathy and skeletal muscle remodeling, resulting in a poor endurance muscle performance (Cluley and Cochrane, 2001; Turk et al., 2017). In this case, exercise can be limited more by leg fatigue than by dyspnea so that some patients prefer to reduce their activity (Barreiro et al., 2004). As described in the GINA document, symptoms control, reduction in future risk, and improvement in QoL are the primary goal of asthma treatment. Pharmacological treatment included inhaler corticosteroids and/or bronchodilators and other oral controllers according to constantly updated national and international guidelines. Over past few years, some studies have introduced the role of pulmonary rehabilitation as an additional nonpharmacologic therapy.

In 2013, the American Thoracic Society and the European Respiratory Society created a document in support of this type of intervention. In details, they described Pulmonary Rehabilitation as a “comprehensive intervention based on a thorough patient assessment followed by patient tailored therapies that include, but are not limited to, exercise training, education, and behaviour change, designed to improve the physical and psychological condition of people with chronic respiratory disease and to promote the long-term adherence to health-enhancing behaviours” (Spruit et al., 2013; Nici and ZuWallack, 2014). So far, there has been a robust literature regarding indications and components of pulmonary rehabilitation in patients affected by chronic obstructive pulmonary disease (COPD) with positive results in terms of both quality of life and exercise tolerance. However, similar aspects have not been completely documented and clarified for asthmatic patients, resulting in increasing interest.

Pulmonary rehabilitation is a multidisciplinary approach of disease management and involves doctors, respiratory therapists, nurses, psychologists, and dieticians. Usually the patient undergoes a comprehensive assessment by specialist in order to tailor each interventions on specific patient’s needs; therefore, a preliminary assessment should be performed. There is not a universal program applicable on every patient. Indeed, each program may vary because it should take into account the presence of comorbidities, such as cardiac, musculoskeletal, metabolic, and neurologic diseases, that may affect both exercise performance and quality of life.

It usually consists of education, exercise training, breathing retraining, and psychological support.

## Education

Educational support has always been considered a key feature in the management of patients affected by respiratory diseases. Education about lung disease and its management has long been a precious component of pulmonary rehabilitation. In a systematic review, Normansell highlighted that adherence to inhaler treatment in asthmatic patients is challenging and it constitutes a persistent barrier to treatment; it has been estimated that adherence in asthma population ranged from 47 to 57% (Normansell et al., 2017) which is even lower than in other chronic diseases, such as rheumatologic or gastrointestinal diseases (Di Matteo, 2004). Non-adherence to pharmacological treatment could be multifactorial, in most of the cases patients underestimate their symptoms and are not completely aware of their disease and therefore do not understand the importance of taking medications. In addition, as for other respiratory diseases, also for asthma, treatment is based on inhalers through different devices which require collaborations by patients.

Education implies that specialists teach patients about respiratory diseases and support them through self-management training. This approach includes the use of motivational interviewing with the support of social media and web-based applications. This method seems to improve clinical practice and allows patients to share their doubts and to practice the correct techniques for using inhalers and nebulizers (Kew et al., 2016; Yorke et al., 2017; Schuermans et al., 2018; Gesinde and Harry, 2018). Knowledge about the disease helps patients to understand, recognize, and treat the symptoms and therefore to achieve a better asthma control in daily life (Spruit et al., 2013; Boudreau et al., 2014; Levy et al., 2016; Donner et al., 2018). It could be a single short intervention, typically 10 min or standardized (i.e. 30 min through booklet, videos one to one or in a group) on useful topics, including smoking cessation, oxygen therapy, nutrition, physical activity, proper use of medications, and health preservation (e.g. vaccinations). The smart technologies could be used to empower patients in asthma management and help to remind patients the optimal timing to take medications and how to take them. Trackable data could help clinicians to detect problems and to support patients addressing any difficulties. In a recent review by Farzandinpour et al. (2017), 10 studies were evaluated to summarize the evidences regarding the effects of mobile health applications for self-management outcomes. Authors concluded that multifunctional apps can improve asthma control and the quality of life of patients compared to traditional interventions. However, further studies are needed in order to identify the effectiveness of these interventions on the outcomes related to medication adherence and costs. Nevertheless taken alone, these reminder systems are effective in a short-term period (Normansell et al., 2017; Gesinde and Harry, 2018). Indeed, asthma patients need to be supported to achieve health goals; however, health professionals can rely on these multidimensional approaches only when trained to use them.

## Exercise Training

The cornerstone of pulmonary rehabilitation is exercise training. There is evidence supporting a positive role of exercise training in improving exercise performance in asthmatic patients; however, as asthma ranges widely, specific programs should be tailored on patient’s characteristics (Satta, 2000). First of all patient should undergo a complete lung function assessment, including spirometry before and after bronchodilator and exercise capacity testing. Since a substantial proportion of patients with asthma experience EIB, it’s important to diagnose and guide health care providers with regard to the management and treatment of EIB.

Exercise capacity is usually based on cardiopulmonary exercise testing or a field tests as 6-min or shuttle walk test. There is no universal agreement on the type of exercise training in respiratory diseases; it can vary from endurance training or strength training. So far, the former is the most studied in clinical trials and the most common exercise used in pulmonary rehabilitation program worldwide.

## Endurance Training

It involves upper and lower extremities and the workload should increase as the patient’s performance improves. It is typically scheduled two to five times a week over 3–8/12 weeks. For patients with EIB, according to the current guidelines, short-acting b2-agonist should be administered 15 min before starting exercise (Spooner et al.,; Global Initiative for Asthma, 2018). The training workload is based on a cardiopulmonary exercise test or field walking test (respectively at 60–70% VO2 max or 60–80–100% HR max) with continuous or interval exercise for 20–30 min (Maltais et al., 1997; Luxton et al., 2008; Borg et al., 2010; Spruit et al., 2013; Lingner et al., 2015; Turk et al., 2017; Toennesen et al., 2018). The mechanism by which exercise improves endurance remains unclear. However, several studies have proven that exercise training strengthens peripheral muscles leading to biological and physiological changes that lower respiratory rate, ventilatory requirement and reduce dynamic lung hyperinflation (Porszasz et al., 2005), in addition to a psychological training effect. To date, there are few data available concerning baseline patients characteristics that can predict who could benefit from pulmonary rehabilitation, especially when stratified by disease severity. In parallel with COPD patients, also asthmatic patients with more severe impairment and poor diseases control are more limited in daily life activities (Global Initiative for Asthma, 2018). A retrospective study by Zampogna et al. (Zampogna et al., 2019) showed that asthmatic individuals belonging to any GINA step benefit from a pulmonary rehabilitation program in terms of respiratory symptoms, muscle fatigue, and oxygen value at rest and exercise performance. In addition, younger patients, with smoking history and worse baseline exercise tolerance seemed to benefit from pulmonary rehabilitation (Zampogna et al., 2019). Zampogna et al. (Zampogna et al., 2019) performed a similar analysis on a selected population of 317 patients affected from severe asthma and observed that a multidisciplinary pulmonary rehabilitation program was effective in terms of exercise capacity, assessed through the 6-min walking test, and respiratory symptoms, assessed with Borg fatigue and dyspnea (Zampogna et al., 2019). There is evidence in the literature about the benefits of pulmonary rehabilitation in patients with partially or uncontrolled asthma. Sahin and Naz (2019) observed the effects of an 8-week outpatient pulmonary rehabilitation program on respiratory symptoms, comparing 21 patients with partially controlled to 28 with uncontrolled asthma. The results documented that there was a better improvement in latter highlighting the potential beneficial effect of pulmonary rehabilitation in selected asthmatic patients (Sahin and Naz, 2019). As previously mentioned, asthma is characterized by an underline chronic airway inflammation that causes an exaggerated contractile response of the airways to a variety of stimuli. A prospective randomized trial by Franca-Pinto et al. (2015) studied the effect of exercise as an add on nonpharmacological treatment on 58 moderate–severe asthma patients randomly assigned to either the control group or the aerobic training group and followed over a 3-month period. The results demonstrated that pulmonary rehabilitation had a positive effect on bronchial hyperresponsiveness, serum inflammation, QoL, and asthma exacerbations. In addition, a systematic review and meta-analysis performed by Eichenberger et al. (2013) documented that physical activity on a regular basis may be beneficial on respiratory symptoms, quality of life, and bronchial inflammation.

## Strength Training

Strength training improves muscle mass and strength. Patient is asked to perform a set of repetitive exercises involving peripheral muscle, upper and lower extremities, starting with or without weights tailored according to previous 1 Repetition Maximum Strength test. Patients usually start with eight repetitions and progress up to 10. Successively the load should be increased by 2–10%, when the patient is able to perform the workload for one to two repetitions above the ideal number (Puhan et al., 2006). The training frequency ranges from three to five weekly sessions for a total amount of 15–20. Potential benefit is that resistance exercise involves single muscle groups and results in lower oxygen consumption and minute ventilation and therefore it provides additive benefit to endurance training. To our knowledge, preliminary data that should be confirmed, on the effects of strength training in asthmatic patients are available (Olenich et al., 2018). Even if research data support a promising role of exercise in asthma, little is known about patient’s characteristics that can predict the beneficial effect of rehabilitation program in asthma.

## Physical Activity

Physical activity may influence asthma incidence. In a systematic review and meta-analysis Eijkemans et al. highlighted that physical activity may be a promising protective factor against the development of asthma (Eijkemans et al., 2012). However, data in literature available so far are conflicting. Garcia-Aymerich et al. documented that physically active women have got a lower risk of asthma exacerbations (Garcia-Aymerich et al., 2009) whereas Brumpton et al. did not find significant improvement (Brumpton et al., 2016). Furthermore, there is a strong association between body mass index and the risk of developing poorly controlled asthma, so it could be expected that low levels of physical activity may contribute to increase the same risk as well. Higher adherence to physical activity is associated with favorable clinical outcomes (Panagiotou et al., 2020). Patients should be invited, if no contraindications, to maintain an active lifestyle, such as suggested by World Health Organization “at least 150 minutes of moderate-intensity physical activity throughout the week, or do at least 75 minutes of vigorous-intensity physical activity throughout the week, or an equivalent combination of moderate- and vigorous-intensity activity.” The intensity of physical activities is different between people. All activities must be performed in periods of at least 10 min in order to be beneficial for cardiorespiratory system (https://www.who.int/news-room/fact-sheets/detail/physical-activity). The results of a systematic review suggest that the use of a pedometer is associated with significant increase in physical activity in adult population. However, the use of pedometer in asthma patients is still poorly studied. In a randomized study by Coelho et al., clinically stable adults with moderate to severe asthma were asked to take part into a unsupervised pedometer-based physical activity program; compared to the control group, the experimental group showed a significant difference in terms of daily steps between at 12 weeks, but not at 24–28 weeks (Coelho et al., 2018).

## Breathing Retraining

Breathing retraining is considered an alternative exercise training being a less conventional form of exercise and its role in pulmonary rehabilitation is not completely clear. Retraining with breathing techniques aims to reduce respiratory rate and improve ventilation and gas exchange in order to reduce air trapping. Diaphragmatic breathing increases tidal volume, but studies so far have produced conflicting results in COPD (Gosselink et al., 1995; Yamaguti et al., 2012). Inspiratory muscle training has been shown to decrease dyspnea, increase inspiratory muscle strength, and improve exercise capacity in asthmatic subjects (Duruturk et al., 2018). So far, there is no definitive evidence to support or avoid inspiratory muscle training for asthma (Silva et al., 2013; Shei et al., 2016), therefore, specific respiratory muscle tests and training should be limited to patients carefully selected by clinicians taking into account clinical history.

## Breathing Exercises

Asthmatic patients with not well controlled respiratory symptoms may benefit from breathing exercises (Bruton et al., 2018). Up to 30% of patients report that they use breathing techniques to control their symptoms (Ernst, 2000). Usually, the exercises comprise training in nasal and slow breathing, controlled breath holds and relaxation exercises and should be administered face to face by a respiratory therapist or self-guided by digital supports (Nickel et al., 2005; Bruton et al., 2018). A new treatment’s option which seems to be in line with breathing exercises is yoga. Yang et al. carried on a study showing positive effects of yoga on quality of life and symptoms in patients affected from asthma (Yang et al., 2016).

## Nutritional Counseling And Weight Management

Rehabilitation programs should include nutritional counseling and weight management. Patients with lung disease are at risk for obesity because of limitations to physical activity and adverse effects of oral glucocorticoids given for exacerbations; weight loss can help to reduce the work of breathing. Obesity is an important risk for asthma: it has been estimated that overweight people are 38 more likely to develop asthma than patients with normal weights. Data for obese people is even worse considering that the risk increase up to 92% (Stream and Sutherland, 2012) so that Global Initiative for asthma (Global Initiative for Asthma, 2018) recommends weight loss for all obese asthmatic patients. Indeed, obesity is a common comorbidity of asthma patients and obese patients are at high risk to develop difficult to treat asthma, with poor control of respiratory symptoms and reduced response to corticosteroids (Beuther and Sutherland, 2007; Sutherland, 2014; Freitas et al., 2017). Exercise and weight loss programs are effective treatments for non-obese asthma patients also (Toennesen et al., 2018; Ricketts and Cowan, 2019; Zampogna et al., 2019).

## Psychological Counseling

Asthma is a risk factor for the development of anxiety and depression and may contribute to fatigue and reduce physical activities. Psychological counseling should be offered either individually or in small groups to adults with persistent asthma because discussion will enable patients to feel more comfortable with their disease and incline to participate in social activities. In details, cognitive behavioral therapy is a form of talking therapy that explores a person’s perceptions of themselves and others and how a person’s behavior influences their thoughts and feelings. Cognitive behavioral therapy may improve QoL, asthma control, and anxiety levels compared with usual care (Yorke et al., 2017).

## Conclusion

Although evidence supports a well-established role of pulmonary rehabilitation in patients with COPD, the optimal duration of the activity, frequency, and intensity of the exercise sessions and specific characteristics of specialist’s interventions in asthma patients remain poorly defined. Pulmonary rehabilitation seems to improve exercise tolerance, respiratory symptomsquality of life in asthma patients (the main interventions are summarized in **Table 1**). Recent studies have highlighted that patients with severe symptoms are likely to be the ones who benefit the most from rehabilitation programs with additional improvement also in airways inflammation. The aim of asthma treatment is to improve symptoms and reduce future risk. In this contest, comorbidities in asthmatic patients like in other chronic respiratory diseases with lung impairment (Visca et al., 2013; Visca et al., 2019) play a key role and could negatively interfere in the whole management of the disease. Preliminary data show that obesity, sleep apnea syndrome, and bronchiectasis may benefit from pulmonary rehabilitation in asthma patients (Zampogna et al., 2019). In conclusion, further studies are needed to address the role of pulmonary rehabilitation and specific programs, both hospital and home settings in order to improve clinical care in asthma.

**Table 1:** Summary of main pulmonary rehabilitation interventions and outcomes.

INTERVENTION	STUDY	OUTCOMES
Education	(Boudreau et al., 2014; Kew et al., 2016; Levy et al., 2016; Farzandipour et al., 2017; Normansell et al., 2017; Yorke et al., 2017; Donner et al., 2018; Gesinde and Harry, 2018; Schuermans et al., 2018)	Increased medication technique and adherence Better asthma control
Exercise	(Maltais et al., 1997; Porszasz et al., 2005; Luxton et al., 2008; Borg et al., 2010; Eichenberger et al., 2013; Spruit et al., 2013; Franca-Pinto et al., 2015; Lingner et al., 2015; Turk et al., 2017; Toennesen et al., 2018; Sahin and Naz, 2019; Zampogna et al., 2019; Zampogna et al., 2019)	Increased muscular endurance and strength Improved QoL Reduced bronchial inflammation, asthma symptoms, and exacerbations
Physical activity	(Garcia-Aymerich et al., 2009; Eijkemans et al., 2012; Coelho et al., 2018; Panagiotou et al., 2020; Physical Activity On, 2020)	Reduced risk of development asthma
Breathing retraining	(Gosselink et al., 1995; Yamaguti et al., 2012; Duruturk et al., 2018)	Increased inspiratory muscle strength Increased exercise capacity Reduced symptoms
Breathing exercise	(Yang et al., 2016; Bruton et al., 2018)	Reduced symptoms
Weight loss	(Beuther and Sutherland, 2007; Stream and Sutherland, 2012; Sutherland, 2014; Freitas et al., 2017; Toennesen et al., 2018; Ricketts and Cowan, 2019)	Better asthma control Reduced symptoms
Psychological counseling	(Yorke et al., 2017)	Improved QoL Reduced anxiety

## Author Contributions

EZ, DV: conception of the work and interpretation of data. EZ, MZ: drafting the work. EZ, DV, AS: revising it critically for important intellectual content.

## Conflict of Interest

The authors declare that the research was conducted in the absence of any commercial or financial relationships that could be construed as a potential conflict of interest.
